# Research on Medical Image Segmentation Based on SAM and Its Future Prospects

**DOI:** 10.3390/bioengineering12060608

**Published:** 2025-06-03

**Authors:** Kangxu Fan, Liang Liang, Hao Li, Weijun Situ, Wei Zhao, Ge Li

**Affiliations:** 1 School of Computer Science and Engineering, Central South University, Changsha 410083, China; 2 Department of Radiology, The Second Xiangya Hospital, Central South University, Changsha 410011, China; 3 Department of Radiology, Xiangya Hospital, Central South University, Changsha 410008, China

**Keywords:** Segment Anything Model, medical image segmentation

## Abstract

The rapid advancement of prompt-based models in natural language processing and image generation has revolutionized the field of image segmentation. The introduction of the Segment Anything Model (SAM) has further invigorated this domain with its unprecedented versatility. However, its applicability to medical image segmentation remains uncertain due to significant disparities between natural and medical images, which demand careful consideration. This study comprehensively analyzes recent efforts to adapt SAM for medical image segmentation, including empirical benchmarking and methodological refinements aimed at bridging the gap between SAM’s capabilities and the unique challenges of medical imaging. Furthermore, we explore future directions for SAM in this field. While direct application of SAM to complex, multimodal, and multi-target medical datasets may not yet yield optimal results, insights from these efforts provide crucial guidance for developing foundational models tailored to the intricacies of medical image analysis. Despite existing challenges, SAM holds considerable potential to demonstrate its unique advantages and robust capabilities in medical image segmentation in the near future.

## 1. Introduction

Medical image segmentation plays a pivotal role in clinical applications, ranging from diagnostic imaging to treatment planning. While deep learning models have increasingly dominated this field, a persistent challenge lies in the need to train distinct models for each segmentation task [1,2,3], such as delineating brain structures, cardiac anatomy, or prostate boundaries. This requirement imposes substantial resource burdens, particularly in acquiring high-quality annotated datasets with precise ground truth masks.

The emergence of the Segment Anything Model (SAM) [4] has thus attracted significant attention. SAM’s promptable design and foundation on large-scale image pretraining enable it to generalize across domains without additional fine-tuning, suggesting transformative potential for medical image processing. Unlike conventional segmentation networks that require extensive labels and retraining for each new organ or modality, SAM can produce accurate masks in a zero-shot manner by leveraging user interactions (e.g., point clicks, bounding boxes). This feature could drastically reduce annotation time and cost in clinical workflows.

This paper systematically reviews SAM’s applicability to medical image segmentation. We first outline SAM’s architecture and operational principles. Next, we evaluate SAM’s performance against state-of-the-art algorithms designed explicitly for medical imaging tasks. Our analysis focuses on two key aspects: (1) benchmarking SAM across diverse medical segmentation tasks and imaging modalities, and (2) exploring methodological enhancements to improve SAM’s adaptability to these tasks.

**Figure 1:**
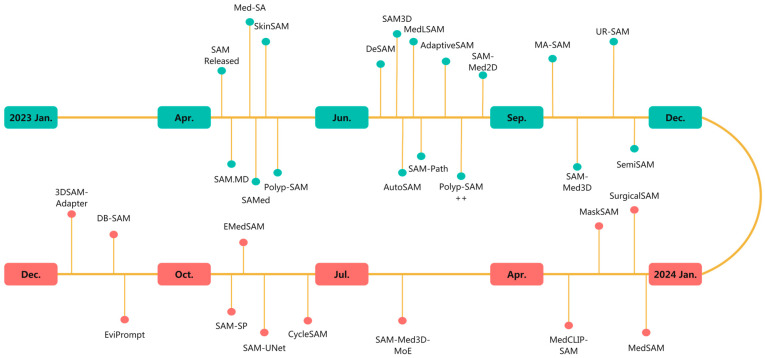
A brief timeline of SAM and its variants applied in medical image segmentation.

In Figure 1, we plot a timeline of various variants and applications of SAM in medical image segmentation tasks from January 2023 to December 2024.

## 2. The Architecture of SAM

**Figure 2:**
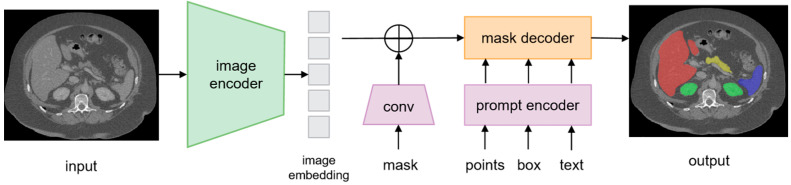
Overview of the Segment Anything Model (SAM) architecture.

The Segment Anything Model (SAM) was trained on the largest segmentation dataset to date, comprising over 1 billion ground truth masks derived from 11 million licensed, privacy-protected natural images [4]. Upon its release, SAM demonstrated exceptional zero-shot generalization across 23 diverse natural image datasets, surpassing both interactive and task-specific models without requiring retraining or fine-tuning. SAM’s architecture integrates three core components (Figure 2). The model employs an image encoder to extract rich image embeddings, incorporates user interactions via a prompt encoder that supports various prompt types, and generates segmentation masks through a mask decoder by effectively fusing image and prompt embeddings. This architecture enables flexible and prompt-driven segmentation, facilitating generalization across diverse scenarios without the need for retraining.

Image Encoder: A Vision Transformer (ViT)-based [5] encoder pre-trained using the Masked Autoencoder (MAE) [6] methodology. This encoder processes 1024 × 1024-pixel inputs, generating a 64 × 64 feature map (downscaled by a factor of 16). Its design prioritizes scalability and compatibility with high-resolution images.

Prompt Encoder: SAM supports sparse prompts (e.g., points, bounding boxes) and dense prompts (e.g., masks). Sparse prompts are encoded using positional embeddings and trainable tokens for foreground/background differentiation. Bounding boxes are represented by their upper-left and lower-right corner coordinates. Dense prompts retain input resolution and are processed via convolutional embeddings combined with image features.

Mask Decoder: A lightweight decoder incorporating two transformer [7] layers, a dynamic mask prediction head and an Intersection-over-Union (IoU) regression head. The mask head produces three outputs at 4× scaling: whole-object, part-level, and sub-part segmentations.

Training employs a hybrid loss function combining focal loss [8] and dice loss [9], optimized for promptable segmentation tasks. To enhance label efficiency, SAM’s development utilized a three-stage data engine: (1) interactive mask annotation by experts, (2) manual correction of under-segmented regions, and (3) automated selection of stable pseudo-masks for final training.

## 3. Evaluation of the Zero-Shot Capability of the Segment Anything Model (SAM) in the Field of Medical Image Segmentation

### 3.1. Evaluation on Specific Datasets

#### 3.1.1. CT Image Segmentation

Computed tomography (CT) generates cross-sectional anatomical images by capturing X-ray projections from multiple angles. These images enable detailed visualization of internal structures, including organs, bones, and pathologies. To assess SAM’s zero-shot capability for abdominal organ segmentation, Roy et al. [10] evaluated SAM on the AMOS22 [11] dataset using randomly sampled points and jittered bounding boxes to simulate user variability. Their results revealed that SAM underperformed compared to state-of-the-art (SOTA) methods when using point prompts, with Dice Similarity Coefficients (DSCs) declining by 20.3–40.9%. However, SAM achieved competitive accuracy with bounding box prompts under moderate jittering. For tumor segmentation, Hu et al. [12] tested SAM on contrast-enhanced CT (CECT) volumes of multi-phase liver tumors. While increasing the number of prompt points improved SAM’s performance, a substantial gap persisted relative to the classical U-Net [13] architecture, underscoring limitations in handling complex, low-contrast lesions.

#### 3.1.2. Colonoscopic Image Segmentation

Colonoscopy captures high-resolution images of the colonic mucosa to detect abnormalities such as polyps and inflammatory lesions. Zhou et al. [14] evaluated SAM’s polyp segmentation performance across five benchmark datasets. In prompt-free settings, SAM exhibited markedly reduced accuracy (DSC decline: 14.4–36.9%) compared to SOTA methods. This deficiency was attributed to SAM’s difficulty in distinguishing subtle boundaries between polyps and surrounding mucosa, highlighting the need for adaptive prompts or auxiliary techniques.

**Figure 3:**
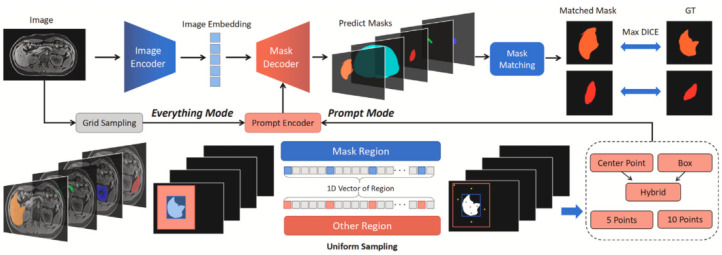
Zero-shot evaluation pipeline of SAM in medical image segmentation tasks [15].

As shown in Figure 3, the pipeline consists of three core components: an image encoder, a prompt encoder, and a mask decoder. Given an input medical image (e.g., CT or MRI), the image encoder extracts high-dimensional visual features. In Everything Mode, the model performs grid-based sampling across the entire image to automatically generate a set of candidate masks. In contrast, Prompt Mode utilizes either simulated or manually selected prompts derived from ground-truth annotations, such as points, bounding boxes, or mask regions. These prompts are embedded through the prompt encoder. The mask decoder then integrates both the image features and prompt embeddings to predict segmentation masks. For performance evaluation, a mask matching module selects the best-matched predicted mask for each ground-truth region using the maximum Dice similarity score.

### 3.2. Evaluation on Multimodal Datasets

He et al. [16] conducted a large-scale empirical analysis of SAM across 12 public datasets spanning diverse organs (brain, breast, lung, liver, etc.) and modalities (2D X-ray, ultrasound, 3D MRI/CT). Without fine-tuning, SAM demonstrated inconsistent accuracy, with performance heavily influenced by image dimensionality, modality, object size, and contrast. Similarly, Mazurowski et al. [17] evaluated SAM on 11 datasets using simulated point prompts. While SAM excelled in segmenting well-defined structures, it struggled with intricate targets like tumors, emphasizing its reliance on precise prompts.

Cheng et al. [18] systematically compared SAM’s three prompt modes (automatic, box, point) across 12 datasets. Box prompts without jittering yielded the highest zero-shot accuracy, though performance varied significantly across tasks. In clinical radiotherapy, Zhang et al. [19] tested SAM on critical treatment sites (prostate, lung, head/neck). SAM effectively segmented large organs but faltered with smaller, complex structures under ambiguous prompts.

To consolidate these findings, Huang et al. [15] constructed COSMOS 1050K—a comprehensive benchmark comprising 52 datasets (18 modalities, 84 anatomical/pathological targets). Evaluations across prompt modes confirmed SAM’s superiority with manual prompts (points/boxes). Notably, introducing negative prompts slightly degraded performance in tasks with ambiguous foreground–background distinctions, underscoring the importance of domain-specific prompt selection.

**Figure 4:**
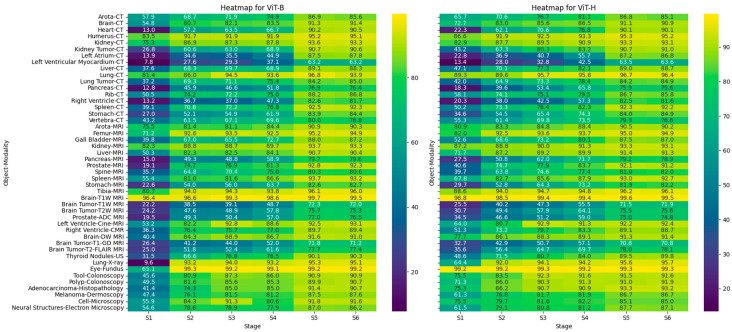
Heatmap of DICE scores (%) for selective common medical objects across different imaging modalities, comparing the small (ViT-B) and large (ViT-H) encoders of the SAM. Each row is an Object–Modality pair; each column S1–S6 corresponds to one of six test strategies.

Huang et al. [15] systematically summarized the DICE scores (%) (Figure 4) for selected common medical structures across diverse imaging modalities, with ViT-B and ViT-H denoting the small and large encoders of the SAM, respectively. Six prompting strategies were evaluated: S1 (Everything Mode), S2 (1-point prompt), S3 (5-point prompt), S4 (10-point prompt), S5 (box prompt without 1-point), and S6 (box prompt with 1-point).

Results show that under Everything Mode, ViT-H achieved a 7.47% improvement in DICE scores over ViT-B. With a 1-point prompt, ViT-H outperformed ViT-B marginally, and the performance gap widened as the number of point prompts increased. In contrast, performance differences between ViT-B and ViT-H were minimal for box-based prompts. Overall, box prompts, by providing richer spatial priors, enhanced SAM’s segmentation performance more effectively than point-based prompts.

### 3.3. Challenges in Low-Contrast Imaging Modalities

Despite SAM’s promising zero-shot capabilities across certain modalities, its performance on low-contrast medical imaging remains particularly concerning. Modalities such as ultrasound and X-ray often lack clear intensity gradients and exhibit high levels of noise, anatomical ambiguity, and operator-dependent artifacts. Studies such as He et al. [16] and Mazurowski et al. [17] reveal that SAM struggles to delineate subtle tissue boundaries or distinguish pathological structures in these conditions. For instance, in ultrasound-based organ segmentation, SAM often misidentifies anatomical landmarks due to speckle noise and poor contrast, while in chest X-ray imaging, overlapping anatomical structures pose significant challenges in accurate mask prediction. These observations highlight a fundamental limitation: SAM’s general-purpose architecture is not inherently robust to modality-specific image degradation and lacks the semantic prior knowledge required to compensate for missing contextual cues. Without modality-adaptive prompt tuning or specialized pre-processing pipelines, SAM remains prone to under- or oversegmentation in low-contrast environments—limiting its clinical applicability in these common diagnostic modalities.

## 4. The Application of Segment Anything Model (SAM) in the Field of Medical Image Segmentation

### 4.1. Fine-Tuning on Medical Image Datasets

#### 4.1.1. Full-Parameter Fine-Tuning

To enhance SAM’s performance in medical tasks, full-parameter fine-tuning has been widely explored. For skin cancer segmentation, Hu et al. [20] demonstrated that fine-tuning SAM increased the DSC from 81.25% to 88.79%. Similarly, Li et al. [21] developed PolypSAM, a SAM variant optimized for polyp segmentation, achieving DSC scores >88% across five public datasets.

A notable advancement is MedSAM [22], a generalized medical segmentation model trained on 11 imaging modalities and >1 million image–mask pairs. MedSAM achieved median DSCs of 94.0% (intracerebral hemorrhage CT), 94.4% (glioma MR T1), 81.5% (pneumothorax XR), and 98.4% (polyp endoscopy), outperforming specialized U-Net [13] models. However, MedSAM struggles with complex structures like blood vessels, primarily due to the ambiguity of bounding box prompts and its reliance on 2D slice-based processing for 3D volumetric data, which fails to capture the intricate 3D relationships and fine details of such structures. This limitation hinders the model’s ability to accurately segment the more complex, branched morphology of blood vessels.

#### 4.1.2. Parameter-Efficient Fine-Tuning (PEFT)

**Figure 5:**
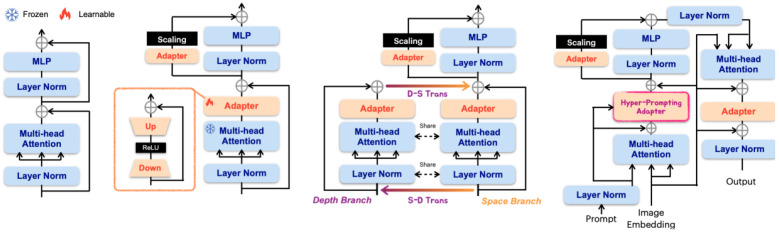
The SAM Medical Adapter (Med-SA) [2] adapts SAM to medical image segmentation in a parameter-efficient manner.

Full-parameter updates are computationally prohibitive for clinical deployment. To address this, researchers have adopted PEFT techniques. Wu et al. [2] introduced the Medical SAM Adapter (Med-SA) (Figure 5), integrating Low-Rank Adaptation (LoRA) [23] modules into SAM without altering its pre-trained weights. Med-SA performs well because it efficiently incorporates domain-specific medical knowledge and adapts SAM to the 3D nature of medical images using Space-Depth Transpose (SD-Trans), while Hyper-Prompting Adapter (HyP-Adpt) enables prompt-conditioned adaptation, improving its segmentation performance with minimal parameter updates. Evaluated across 17 tasks spanning five modalities, Med-SA surpassed both SAM and prior SOTA methods.

Similarly, SAMed [24] extended LoRA to SAM’s image encoder and fine-tuned the prompt encoder/mask decoder using the Synapse dataset. With only 0.1% of SAM’s parameters updated, SAMed achieved a DSC of 81.88%, matching SOTA performance. Feng et al. [25] combined example-guided synthesis with LoRA to align SAM with medical data under limited annotations.

Paranjape et al. [26] proposed AdaptiveSAM, an adaptive framework that enhances SAM’s adaptability to new medical datasets and enables text-prompt-driven segmentation. By incorporating bias adjustment mechanisms, AdaptiveSAM achieves a significant reduction in trainable parameters compared to the original SAM while utilizing free-form text prompts for precise object delineation. Experimental evaluations across diverse medical imaging datasets—including surgical, ultrasound, and X-ray modalities—demonstrate that AdaptiveSAM outperforms existing state-of-the-art methods in accuracy and generalizability.

To address the significant domain disparity between natural and medical images, Cheng et al. [27] introduced SAM-Med2D, a pioneering framework designed to optimize SAM for 2D medical imaging. This methodology enhances the prompt encoder through interactive training and augments the mask decoder by integrating learnable adaptation layers into the image encoder. The authors constructed a large-scale medical segmentation dataset comprising 4.6 million images and 19.7 million annotated masks. Rigorous evaluations across diverse modalities, anatomical regions, and clinical scenarios—including nine MICCAI 2023 challenge datasets—demonstrated SAM-Med2D’s superior segmentation accuracy and robust generalization capabilities compared to the original SAM.

#### 4.1.3. Trade-Offs Between Full Fine-Tuning and PEFT

The decision between full-parameter fine-tuning and parameter-efficient fine-tuning (PEFT) represents a critical trade-off between segmentation accuracy and computational resource consumption. Full-parameter fine-tuning, as demonstrated by Hu et al. [20] in skin cancer segmentation (with a DSC improvement from 81.25% to 88.79%) and Li et al. [21] in polyp segmentation (achieving DSCs exceeding 88% across multiple datasets), offers superior performance by optimizing all model parameters to capture domain-specific features. However, this approach demands substantial computational resources, particularly when applied to large foundational models like the SAM. Consequently, full-parameter fine-tuning is often impractical for clinical deployment, especially in settings with limited hardware capabilities.

In contrast, PEFT methods such as Med-SA [2] and SAMed [24] achieve comparable performance—such as the 81.88% DSC on the Synapse dataset—by updating only 0.1–1% of model parameters. This significant reduction in the number of trainable parameters leads to a considerable decrease in GPU memory usage and training time, making PEFT a more feasible option for real-world applications, particularly in resource-constrained environments.

Recent research further reinforces the benefits of PEFT, confirming that it provides a favorable balance between accuracy and computational efficiency. For instance, Xie et al. [28] demonstrated that their approach could achieve up to 97.6% IoU for femur segmentation using as few as 5–20 labeled images, substantially reducing both GPU memory and training time when compared to full-parameter fine-tuning. Similarly, Paranjape et al. [29] introduced S-SAM, a method that fine-tunes only 0.4% of SAM’s parameters yet still achieves an IoU of 0.84 on the LiTS dataset, outperforming other methods while using far fewer resources. These findings highlight how PEFT can maintain high segmentation accuracy while dramatically lowering computational demands, positioning it as a highly practical solution for medical imaging tasks.

### 4.2. Automated Prompt Mechanism

#### 4.2.1. Automated Prompt Generation

**Figure 6:**
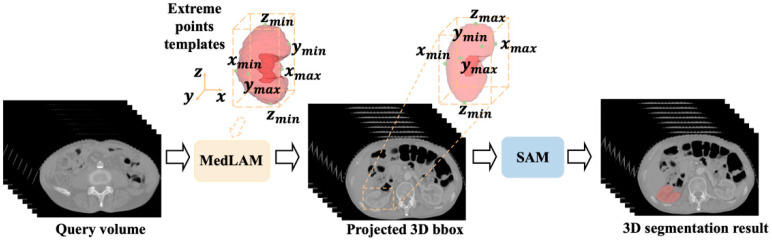
The process of MedLSAM automatically generating prompt boxes in 3D medical image segmentation [32].

Automating prompt generation for SAM can be effectively achieved through localization frameworks. Pandey et al. [30] innovatively integrated the YOLOv8 [31] model to detect Regions of Interest (ROIs) in medical images, generating precise bounding boxes as input prompts for the SAM. This approach enables fully automatic segmentation across diverse imaging datasets. Building on this, MedLSAM [32] introduces a few-shot 3D localization technique that identifies anatomical structures by leveraging spatially consistent pixel distributions. By projecting 3D bounding boxes onto 2D slices, MedLSAM (Figure 6) provides explicit guidance for the SAM, streamlining segmentation of complex anatomical targets. Anand et al. [33] further advanced this paradigm with a one-shot framework that harnesses template image correspondence. A pre-trained Vision Transformer (ViT) [5] extracts dense feature representations from templates, which are then utilized to generate context-aware prompts for the SAM. This method significantly enhances segmentation accuracy while minimizing manual intervention.

#### 4.2.2. Learnable Prompts

Recent advancements in automated prompt engineering for SAM include AutoSAM [34], which introduces an auxiliary prompt encoder to generate conditional prompts directly from input images. This encoder eliminates the need for SAM fine-tuning by extracting image-derived features as contextual guidance, enabling fully automatic segmentation. AutoSAM achieves SOTA performance across multiple medical benchmarks, underscoring its efficacy in complex segmentation tasks. The all-in-SAM [35] framework leverages weak prompts from the pre-trained SAM to generate preliminary pixel-level annotations. These annotations are then used to refine SAM via targeted fine-tuning strategies. This approach removes dependency on manual prompts during inference, surpasses prior SOTA methods in nuclear segmentation, and maintains competitiveness even when compared to models trained on extensively annotated datasets. Gao et al. [36] proposed DeSAM (Decoupling Segment Anything Model) to address the coupling between suboptimal prompts and segmentation accuracy. DeSAM decouples SAM’s mask decoder into two specialized modules: the Prompt-Relevant IoU Module (PRIM), which generates prompt-dependent mask embeddings, and the Prompt-Invariant Mask Module (PIMM), which integrates image embeddings with PRIM outputs to produce final masks. Extensive experiments demonstrate that DeSAM enhances SAM’s robustness to domain shifts in clinical environments, achieving an average improvement of 8.96% in the DSC.

Yue et al. [37] further advanced this domain with SurgicalSAM, a lightweight adaptation that integrates surgical-specific knowledge into SAM through contrastive prototype learning. By refining a prototype-based class prompt encoder, SurgicalSAM achieves precise class-aware segmentation with minimal parameter updates. Evaluations on two public surgical datasets confirm its SOTA performance, highlighting its efficiency and generalizability.

#### 4.2.3. Enhance Robustness to Uncertain Prompts

**Figure 7:**
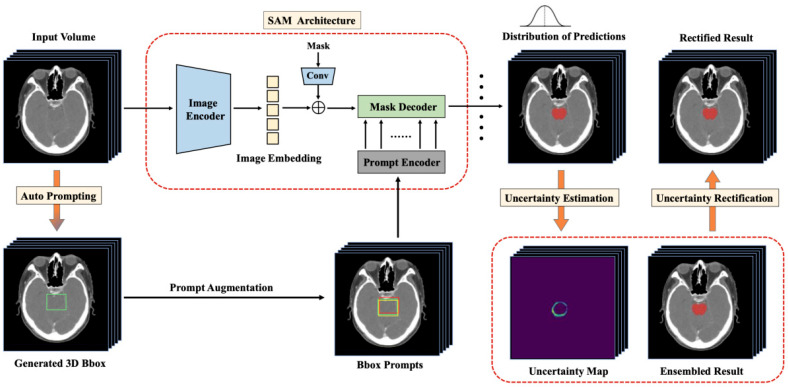
UR-SAM [40] enhances the robustness of automatic segmentation of medical images by estimating and utilizing uncertainties to correct segmentation results.

Given SAM’s sensitivity to input prompts, uncertainty estimation becomes critical to ensuring the reliability of segmentation outcomes—a requirement amplified in medical imaging, where precision directly impacts clinical decision-making. Xu et al. [38] developed EviPrompt, a training-free, uncertainty-driven prompt generation method that automates SAM prompts for medical segmentation without relying on expert input. This approach requires only a single reference image-annotation pair to generalize across tasks. Deng et al. [39] introduced an uncertainty-aware test-time augmentation strategy for SAM in fundus image segmentation. By leveraging multi-box prompts to generate diverse predictions and employing Monte Carlo simulations to estimate uncertainty distributions, their method constructs pixel-wise uncertainty maps to flag potential errors, significantly improving SAM’s prompt robustness. Zhang et al. [40] proposed UR-SAM (Figure 7), an uncertainty-correction framework that refines SAM’s outputs using estimated uncertainty maps. Evaluated on two public 3D datasets (35 organs), UR-SAM achieved DSC improvements of 10.7% and 13.8% without manual prompts, demonstrating its ability to enhance segmentation accuracy while maintaining clinical practicality.

Collectively, integrating uncertainty estimation fortifies SAM’s resilience to prompt variability. Beyond error detection, quantified uncertainty provides clinicians with actionable insights, elevating the trustworthiness of automated segmentation and broadening its applicability in medicine.

### 4.3. Architecture Modification

#### 4.3.1. Synergistic Effects of Training Segmentation Models

Zhang et al. [41] introduced the SAM-Path framework, a fine-tuning mechanism tailored for the SAM to enhance semantic segmentation in digital pathology. This framework incorporates trainable class prompts for targets of interest and employs a pre-trained pathology encoder to infuse domain-specific knowledge, addressing the limitations of comprehensive pathology datasets in SAM training. Their experiments on the CRAG dataset revealed a significant relative improvement of 27.52% in the DSCscore when compared to the original SAM with manual prompts. Chai et al. [42] implemented a stepwise fine-tuning approach that integrated an auxiliary CNN encoder into the standard SAM architecture. This method focused on fine-tuning only the additional CNN and the SAM decoder, thereby reducing computational resources and training duration. Li et al. [43] proposed nnSAM, a novel framework that integrates the pre-trained SAM model into the nnUNet pipeline as a plug-and-play module. This design leverages SAM’s strong domain-agnostic feature extraction ability while fully inheriting nnUNet’s automated configuration and domain-specific adaptability. Notably, nnSAM addresses a major limitation of SAM—its dependency on manual prompts—by eliminating the need for human interaction during inference, thus enabling fully automatic segmentation. Compared with pure prompt-based SAM and non-prompt-based models like nnUNet, nnSAM shows clear superiority, especially under few-shot training scenarios. Experimental results demonstrate that nnSAM consistently outperforms nnUNet in both DICE and ASD across multiple segmentation tasks (e.g., brain white matter, liver, heart, and lung segmentation), with even greater margins under limited training data conditions. Zhang et al. [44] further proposed SAMAug, a method that leverages segmentation masks produced by SAM to augment the input of widely used medical image segmentation models, such as U-Net. Their experiments on two datasets indicated that while SAM may not produce high-quality segmentation for medical images, the masks and features it generates are still valuable for training improved medical image segmentation models. Qin et al. [45] introduced DB-SAM, an adaptation of the Segment Anything Model (SAM) for high-quality universal medical image segmentation. DB-SAM utilizes a dual-branch architecture consisting of a Vision Transformer (ViT) branch and a convolution branch, working in parallel to capture both high-level and low-level domain-specific features. The ViT branch incorporates channel attention blocks to enhance domain-specific feature extraction, while the convolution branch uses lightweight convolutional blocks to capture shallow features. A bilateral cross-attention block is employed to fuse features from both branches, ensuring effective feature integration. Evaluations on a large-scale dataset with both 2D and 3D medical segmentation tasks demonstrated significant improvements, with DB-SAM outperforming SAM and MedSAM by 8.8% in 3D tasks and also showing notable gains in 2D segmentation.

#### 4.3.2. Promote Efficient Annotation Learning

**Figure 8:**
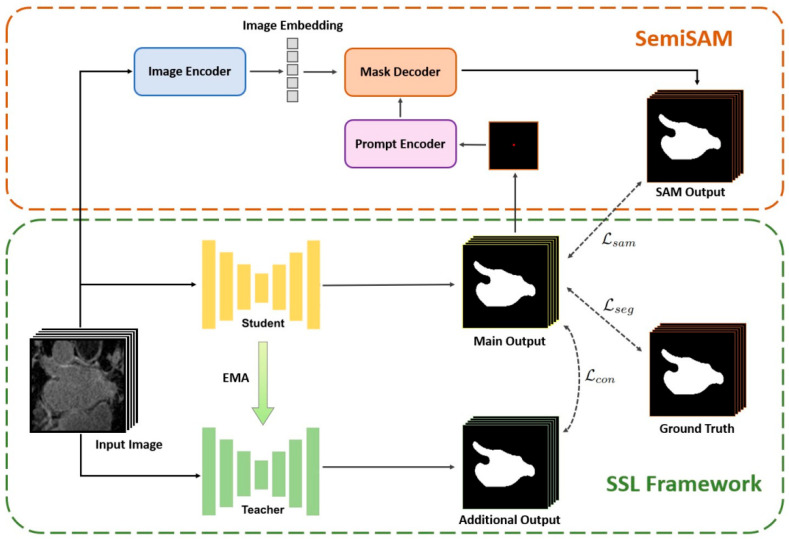
SemiSAM [48] explores the use of the SAM as an additional supervision branch in the process of semi-supervised framework learning.

Due to the elevated costs associated with medical image segmentation, there has been a surge in research focusing on efficient annotation learning techniques, including semi-supervised and weak-supervised learning methods. The SAM has emerged as a reliable pseudo-label generator, facilitating segmentation tasks under scenarios where manually annotated images are scarce. Zhang et al. [46] introduced an iterative semi-supervised method that synergistically integrates the segmentation proposals generated by the SAM with pixel-level and image-level domain-specific knowledge to iteratively construct annotations for unannotated images. In order to generate robust pseudo-labels, Li et al. [47] employed the pre-trained SAM for predictions, aligned these predictions with the generated pseudo-labels, and selectively selected reliable pseudo-labels to further augment the performance of existing semi-supervised segmentation models. They demonstrated improvements of 6.84% and 10.76% on the 5% of annotated data from the publicly available ACDC dataset. Zhang et al. [48] proposed a semi-supervised framework, denoted as SemiSAM (Figure 8), where a segmentation model trained with domain knowledge supplies localization information (i.e., input prompts) to the SAM. In turn, the SAM acts as an additional supervision branch, aiding in consistency learning. Experimental results on the left atrial MRI segmentation dataset indicated that SemiSAM yielded significant improvements under conditions of severely limited annotated data.

### 4.4. Towards 3D Medical Images

#### 4.4.1. Adaptation from 2D to 3D

To facilitate an effective transition from two-dimensional to three-dimensional representations, the Medical SAM Adapter (Med-SA) [2] incorporates the groundbreaking Spatial-Depth Transposition (SDTrans) technique. This technique employs a bifurcated attention mechanism to separately capture spatial and depth correlations within distinct branches.

Notably, Gong et al. [49] developed the 3DSAM Adapter, an optimized iteration of the SAM architecture tailored for volumetric medical image segmentation. Impressively, despite the original model’s tunable parameters accounting for only 16.96% (including the newly introduced parameters), its performance across three datasets substantially outperformed nnU-Net, enhancing renal tumor segmentation accuracy by 8.25%, pancreatic tumor segmentation accuracy by 29.87%, and colon cancer segmentation accuracy by 10.11%, respectively.

Additionally, Chen et al. [50] introduced the Modality-Agnostic SAM Adaptation Framework (MASAM), a versatile framework suitable for various volumetric and video medical data types. The framework astutely integrates tunable 3D adapters within each transformer block of the image encoder and concurrently refines them alongside the mask decoder. Extensive experiments across 10 datasets validated MASAM’s superior performance, consistently outperforming state-of-the-art 3D methods without prompting. Notably, in CT multi-organ segmentation, MRI prostate segmentation, and surgical scene segmentation, MASAM’s Dice Similarity Coefficient (DSC) scores exceeded those of nnU-Net by 0.9%, 2.6%, and 9.9%, respectively.

Li et al. [51] proposed the Prompt-Driven 3D Medical Image Segmentation Model (ProMISe), which inserts lightweight adapters to extract depth-related spatial context without altering pre-trained weights for 3D medical image segmentation. Evaluations on colon and pancreatic tumor segmentation datasets demonstrated ProMISe’s superior performance compared to contemporary state-of-the-art methods.

In conclusion, Bui et al. [52] introduced SAM3D, a novel application of the SAM architecture. SAM3D initially processes each input slice independently, generates slice embeddings, and decodes these embeddings through a lightweight 3D decoder to ultimately produce segmentation results. This approach showcases the innovative potential of SAM in three-dimensional medical image processing.

#### 4.4.2. Training from Scratch

**Figure 9:**
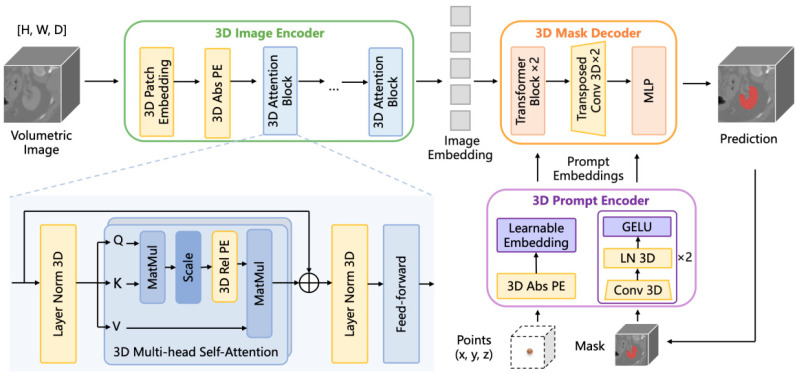
SAM-Med3D [53] converts the original 2D components of SAM into their corresponding 3D versions, including a 3D image encoder, a 3D prompt encoder, and a 3D mask decoder.

Unlike the conventional approach of converting 2D spatial information into 3D, Wang et al. [53] introduced a novel technique, the SAM-Med3D (Figure 9) model. This volumetric medical image segmentation model is grounded in a fully trainable 3D architecture inspired by the SAM. To optimize its performance and robustness, SAM-Med3D was rigorously trained on an extensive 3D dataset encompassing 21,000 medical images and 131,000 corresponding masks. Furthermore, to gauge its practical efficacy, the researchers evaluated SAM-Med3D across 15 distinct public datasets. The results underscored the model’s superior performance and competitiveness within the medical domain. It is particularly noteworthy that SAM-Med3D demands a substantially lower number of prompt points compared to the top fine-tuned SAM models, highlighting its efficiency and practicality.

Building upon the SAM architecture, Du et al. [54] developed an innovative interactive volumetric medical image segmentation model named SegVol, tailored for CT volumetric segmentation. To enhance the model’s capabilities, SegVol was trained on a vast collection of 90,000 unlabeled CT volumes and 6000 labeled volumes. This comprehensive training allows SegVol to achieve precise segmentation across over 200 anatomical categories, leveraging spatial and text-based prompts. Across various segmentation benchmarks, SegVol has demonstrated exceptional performance, outperforming current SOTA methods and showcasing its considerable potential in medical image segmentation.

### 4.5. Comparison Between SAM and Non-Prompt-Based Models

The comparative analysis between the Segment Anything Model (SAM) and traditional models, such as U-Net, has become a key area of research in medical image segmentation. While SAM demonstrates robust feature extraction capabilities, especially when provided with appropriate prompts, its performance tends to diminish when confronted with complex shapes, indistinct boundaries, and high tumor heterogeneity. In contrast, traditional deep learning models like U-Net consistently outperform SAM in these areas, particularly for tasks involving irregularly shaped tumors and those with weak boundaries or high heterogeneity, such as breast tumor and primary brain lymphoma segmentation [55].

To mitigate SAM’s performance degradation in the medical domain, recent works have proposed various strategies, including architectural improvements and fusion with existing models. For instance, SAM-UNet [56] incorporates a U-Net-style convolutional encoder branch into the original SAM framework. This branch is trained independently while the Vision Transformer remains frozen, and a multi-scale fusion mechanism is employed in the mask decoder. Trained on SA-Med2D-16M—the largest 2D medical image segmentation dataset—SAM-UNet achieves state-of-the-art performance, reaching a Dice similarity coefficient of 0.883. More importantly, it significantly improves zero-shot segmentation performance and generalization across unseen modalities. These results highlight the potential of combining convolutional inductive biases with the generalization power of foundation models like the SAM.

In another line of work, the SAM has been utilized to improve existing segmentation pipelines via logit-level fusion. As shown in [57], combining the SAM’s outputs with those from specialized segmentators such as DeepLabv3+ and PVTv2 leads to consistent performance gains across multiple public datasets. By using segmentation masks from mainstream methods as prompts or checkpoints for the SAM and subsequently merging the results, this hybrid approach leverages both the adaptability of the SAM and the domain-specific accuracy of traditional models. Their fusion technique achieved state-of-the-art performance on two challenging datasets (CAMO and Butterfly), showcasing the value of integrating zero-shot foundation models into traditional workflows.

Furthermore, hybrid architectures combining the SAM with domain-specific models like nnU-Net (e.g., SAM + nnUNet and nnSAM) have also demonstrated promising improvements in segmentation accuracy, particularly in low-data regimes and tasks requiring high spatial precision [43]. These findings underscore the importance of selecting and designing model architectures that balance generalization and specificity. While foundation models such as the SAM exhibit considerable promise for prompt-based or zero-shot applications, traditional supervised models like U-Net remain better suited for handling complex and high-stakes medical image segmentation challenges [55,58].

## 5. Discussion

This section outlines the primary challenges, research gaps, limitations, and emerging research directions in the application of the SAM (Segment Anything Model) to medical image segmentation. Despite the SAM’s transformative potential, several critical issues remain, which necessitate continued research to address the gap between its capabilities and the demanding requirements of clinical practice.

### 5.1. Challenges in Achieving Robust Zero-Shot Segmentation

SAM demonstrates impressive performance in certain segmentation tasks, but it struggles with consistency and reliability across different datasets and modalities. This variability arises from the inherent heterogeneity of medical imaging techniques and the complexity of target structures. Medical targets often have an irregular morphology, ambiguous boundaries, small sizes, or low contrast, which complicates segmentation tasks and significantly affects accuracy. These challenges, coupled with the high precision required in clinical settings, make the SAM’s current capabilities inadequate for real-world medical deployment.

To address these limitations, there is a need for continued development of adaptation strategies. Researchers are already exploring techniques like parameter-efficient fine-tuning and uncertainty-aware frameworks that can help bridge the gap between the SAM’s natural image-based training and medical application requirements. However, these strategies have yet to fully match the performance of task-specific models, and further research is needed to enhance the SAM’s robustness.

### 5.2. Domain-Specific Adaptation and Generalization Limitations

To mitigate the domain shift between natural and medical images, researchers have proposed a spectrum of adaptation techniques. These include parameter-efficient fine-tuning (e.g., LoRA), prompt engineering, uncertainty-aware learning, and hybrid learning frameworks. These strategies have enabled the SAM to approach—though not surpass—task-specific segmentation models in terms of accuracy and robustness. Nevertheless, the SAM’s generalist architecture provides a compelling basis for future universal segmentation systems.

Several clinical applications have emerged that underscore the SAM’s translational potential. GazeSAM [59], for instance, integrates the SAM with eye-tracking to support gaze-guided, interactive segmentation, enabling radiologists to generate precise masks with minimal manual effort. Ning et al. [60] demonstrated the SAM’s utility in ultrasound-based navigation systems, enhancing real-time diagnostic accuracy. Similarly, Jiang et al. [61] used the SAM to segment retinal lesions in early-stage diabetic retinopathy, facilitating timely therapeutic intervention. Beyond diagnostic tasks, Song et al. [62] applied the SAM’s semantic priors to improve cross-modal MRI synthesis and image super-resolution, maintaining anatomical fidelity during reconstruction. These examples showcase the SAM’s versatility in both image interpretation and generation, with the potential to reduce manual workload, standardize outputs, and address inter-observer variability—a long-standing issue in medical imaging.

### 5.3. Persistent Challenges and Future Research Directions

Despite promising developments, the SAM’s integration into clinical environments is hindered by several unresolved challenges:

- Computational Overhead: Unlike lightweight, task-specific models designed for deployment efficiency, the SAM’s generalized architecture imposes substantial inference latency and memory costs. These constraints limit real-time applicability, especially in resource-constrained hospital environments. While model pruning, quantization, and hardware acceleration offer possible solutions, these optimizations remain an active area of research.
- Prompt Sensitivity: The SAM’s segmentation results are highly sensitive to variations in prompt type, location, and quality. Even minor prompt changes can cause inconsistent outputs, raising concerns regarding diagnostic reliability. Although the SAM is envisioned as an assistive tool rather than a standalone system, robust prompt engineering protocols and consensus-driven oversight are necessary to ensure consistent, interpretable outputs across users and tasks.
- Dataset Scarcity and Annotation Costs: Foundational models like the SAM rely on vast, high-quality datasets for pretraining and fine-tuning. However, medical image datasets—especially 3D volumetric scans—require expert annotation, which is time-consuming, labor-intensive, and expensive. For comparison, the SAM was trained on SA-1B, which contains 11 million images and 1.1 billion masks [17], whereas constructing medical datasets of this scale is significantly more challenging.

Recent innovations offer potential pathways forward. For example, Liu et al. [63] and Shen et al. [64] integrated various SAM-based models, including FastSAM-3D, into the 3D Slicer platform to enable efficient interactive segmentation of 3D volumetric medical images. Their extension, FastSAM-3DSlicer, supports both 2D and 3D modes, automates raw image handling and mask generation, and features a user-friendly interface that seamlessly integrates into existing workflows. With inference speeds as fast as 0.73 s per volume on GPU and 1.09 s on CPU, it enables near real-time performance. Moreover, their framework introduces uncertainty quantification, improving reliability for clinical use. This interactive tool significantly reduces manual workload and facilitates scalable, high-quality annotation in medical image analysis.

To address these limitations and realize the SAM’s potential in medical settings, the following directions are prioritized:

- Dataset Development: Curate large-scale, domain-specific medical image datasets, particularly 3D volumetric datasets, to enable more effective fine-tuning and benchmarking;
- Hybrid Frameworks: Integrate SAM with lightweight, task-optimized backbones for improved performance on specialized medical tasks;
- Deployment Optimization: Enhance clinical usability through latency reduction, model compression, and inference acceleration to meet real-time processing requirements;
- Prompt Robustness: Establish reliable prompt engineering protocols and develop tools to ensure consistent, reproducible segmentation outputs across users and institutions.

### 5.4. Clinical Feasibility and Ethical Risks

Secondly, ethical considerations arise due to the SAM’s intrinsic prompt sensitivity. Even minor variations in prompt inputs or subtle image changes can generate inconsistent segmentation results, introducing risks in diagnostic reliability. Nevertheless, in realistic clinical scenarios, the SAM is not intended to operate autonomously. Instead, it supports clinicians as an assistive tool, with physicians retaining full authority for diagnostic and therapeutic decision-making. This physician-in-the-loop paradigm substantially mitigates the downstream risk associated with prompt-induced errors, as all outputs are subject to critical clinical oversight. Nonetheless, the adoption of robust consensus guidelines for AI–human collaboration will be essential to safeguard patient safety as the SAM and similar models are integrated into routine practice. Despite these advances, two key considerations—clinical feasibility and ethical risk—warrant careful attention for successful translation of the SAM into high-stakes medical settings.

Firstly, computational feasibility remains a central challenge. Unlike task-specific segmentation models optimized for speed and efficiency, the SAM’s broad, generalized architecture enables diverse applications but incurs a higher computational overhead, often resulting in longer inference times. This trade-off between generalizability and computational efficiency becomes particularly salient in resource-limited hospital environments where inference speed is critical for clinical integration. However, ongoing advancements in algorithms—including model pruning and quantization—as well as greater accessibility to high-performance computing resources, are gradually narrowing this gap. If these technical hurdles can be overcome, SAM’s generalizability may ultimately outweigh the computational costs by obviating the need for multiple task-specific models and streamlining deployment across clinical departments.

### 5.5. Outlook

In summary, the Segment Anything Model represents a foundational shift in the development of general-purpose vision models. Its strengths in scalability and cross-domain generalizability position it as a promising candidate for next-generation clinical tools. However, substantial challenges remain—most notably in precision, efficiency, and domain adaptation. Nevertheless, with focused research efforts across data curation, model optimization, and human–AI interaction design, the SAM has the potential to serve as a transformative component in automated medical image analysis. When coupled with robust clinical oversight and ethically guided integration strategies, the SAM could enable scalable, standardized, and high-fidelity segmentation systems that align with the rigorous demands of modern clinical practice. Methods and open-source resources related to the SAM discussed in this review are comprehensively compiled in Appendix A for reference.

## Data Availability

Not applicable.

## Appendix A

**Table A1:** An initial overview of open source medical segmentation projects related to the SAM.

No.	Method	Title	Code Repository	Paper Link	Description
1	Review	Deep Interactive Segmentation of Medical Images: A Systematic Review and Taxonomy	-	https://ieeexplore.ieee.org/document/10660300 (accessed on 1 May 2025)	Review and taxonomy of deep interactive segmentation in medical images.
2	Med-SA	Medical SAM Adapter: Adapting Segment Anything Model for Medical Image Segmentation	https://github.com/SuperMedIntel/Medical-SAM-Adapter (accessed on 27 March 2025)	https://www.sciencedirect.com/science/article/pii/S1361841525000945 (accessed on 1 May 2025)	Medical SAM adapter for 3D segmentation with minimal parameter updates.
3	SAM	Segment Anything	https://github.com/facebookresearch/segment-anything (accessed on 27 March 2025)	https://openaccess.thecvf.com/content/ICCV2023/papers/Kirillov_Segment_Anything_ICCV_2023_paper.pdf (accessed on 1 May 2025)	Segment Anything Model Origin Paper
9	SAM.MD	SAM.MD: Zero-shot medical image segmentation capabilities of the Segment Anything Model	-	https://openreview.net/forum?id=iilLHaINUW (accessed on 1 May 2025)	Evaluates SAM’s zero-shot medical image segmentation on abdominal CT.
11	Review	When sam meets medical images: An investigation of segment anything model (sam) on multi-phase liver tumor segmentation	-	https://arxiv.org/pdf/2304.08506 (accessed on 1 May 2025)	Evaluating the SAM for multi-phase liver tumor segmentation.
13	SAMPolyp	Can SAM Segment Polyps?	-	https://arxiv.org/pdf/2304.07583 (accessed on 1 May 2025)	Evaluating the SAM for polyp segmentation.
14	Segment-Anything-Model-for-Medical-Images	Segment anything model for medical images?	https://github.com/yuhoo0302/Segment-Anything-Model-for-Medical-Images (accessed on 27 March 2025)	https://www.sciencedirect.com/science/article/pii/S1361841523003213 (accessed on 1 May 2025)	Evaluating the SAM’s performance on medical image segmentation.
17	Review	Sam on medical images: A comprehensive study on three prompt modes	-	https://arxiv.org/pdf/2305.00035 (accessed on 1 May 2025)	Evaluating the SAM’s zero-shot segmentation on medical images with various prompts.
19	SkinSAM	SkinSAM: Empowering Skin Cancer Segmentation with Segment Anything Model	-	https://arxiv.org/pdf/2304.13973 (accessed on 1 May 2025)	Fine-tuned SAM for skin cancer segmentation.
20	Polyp-SAM	Polyp-SAM: Transfer SAM for Polyp Segmentation	https://github.com/ricklisz/Polyp-SAM (accessed on 27 March 2025)	https://www.spiedigitallibrary.org/conference-proceedings-of-spie/12927/1292735/Polyp-SAM-transfer-SAM-for-polyp-segmentation/10.1117/12.3006809.short (accessed on 1 May 2025)	Polyp-SAM adapts the SAM for polyp segmentation, achieving state-of-the-art performance.
21	MedSAM	Segment anything in medical images	-	https://www.nature.com/articles/s41467-024-44824-z (accessed on 1 May 2025)	MedSAM for universal medical image segmentation.
23	SAMed	Customized Segment Anything Model for Medical Image Segmentation	https://github.com/hitachinsk/SAMed (accessed on 27 March 2025)	https://arxiv.org/pdf/2304.13785 (accessed on 1 May 2025)	LoRA-finetuned SAM for medical image segmentation.
24	Fine-tuning SAM on Few Exemplars	Cheap lunch for medical image segmentation by fine-tuning sam on few exemplars	-	https://link.springer.com/chapter/10.1007/978-3-031-76160-7_2 (accessed on 1 May 2025)	Fine-tuning the SAM for medical images with few exemplars.
25	Adaptivesam	Adaptivesam: Towards efficient tuning of sam for surgical scene segmentation	https://github.com/JayParanjape/biastuning (accessed on 27 March 2025)	https://link.springer.com/chapter/10.1007/978-3-031-66958-3_14 (accessed on 1 May 2025)	AdaptiveSAM for efficient surgical image segmentation with text prompts.
26	SAM-Med2D	SAM-Med2D	-	https://arxiv.org/pdf/2308.16184 (accessed on 1 May 2025)	SAM fine-tuned for medical 2D image segmentation with comprehensive prompts.
27	YOLOv8+SAM	Comprehensive Multimodal Segmentation in Medical Imaging: Combining YOLOv8 with SAM and HQ-SAM Models	-	https://openaccess.thecvf.com/content/ICCV2023W/CVAMD/papers/Pandey_Comprehensive_Multimodal_Segmentation_in_Medical_Imaging_Combining_YOLOv8_with_SAM_ICCVW_2023_paper.pdf (accessed on 1 May 2025)	YOLOv8 with the SAM for medical image segmentation.
29	MedLSAM	MedLSAM: Localize and segment anything model for 3D CT images	https://github.com/openmedlab/MedLSAM (accessed on 27 March 2025)	https://www.sciencedirect.com/science/article/pii/S1361841524002950 (accessed on 1 May 2025)	MedLSAM for 3D CT localization and segmentation with minimal annotations.
30	One Shot Localization And Segmentation	One shot localization and segmentation of medical images with Foundation Models	-	https://arxiv.org/pdf/2310.18642 (accessed on 1 May 2025)	One-shot medical image segmentation using natural-image pretrained foundation models.
31	AutoSAM	AutoSAM: Adapting SAM to Medical Images by Overloading the Prompt Encoder	https://github.com/talshaharabany/AutoSAM (accessed on 27 March 2025)	https://arxiv.org/pdf/2306.06370 (accessed on 1 May 2025)	AutoSAM adapts the SAM to medical images via prompt encoder overloading.
32	All-in-sam	All-in-sam: from weak annotation to pixel-wise nuclei segmentation with prompt-based finetuning	-	https://iopscience.iop.org/article/10.1088/1742-6596/2722/1/012012/meta (accessed on 1 May 2025)	All-in-SAM automates nuclei segmentation via prompt-based finetuning from weak annotations.
33	DeSAM	DeSAM: Decoupled Segment Anything Model for Generalizable Medical Image Segmentation	https://github.com/yifangao112/DeSAM (accessed on 27 March 2025)	https://link.springer.com/chapter/10.1007/978-3-031-72390-2_48 (accessed on 1 May 2025)	DeSAM improves the SAM for generalizable medical image segmentation by decoupling prompts and mask generation.
34	SurgicalSAM	SurgicalSAM: Efficient class promptable surgical instrument segmentation	https://github.com/wenxi-yue/SurgicalSAM (accessed on 27 March 2025)	https://ojs.aaai.org/index.php/AAAI/article/view/28514 (accessed on 1 May 2025)	SurgicalSAM enables class-promptable instrument segmentation via prototype-based tuning.
35	EviPrompt	EviPrompt: A Training-Free Evidential Prompt Generation Method for Segment Anything Model in Medical Images	https://github.com/SPIresearch/EviPrompt (accessed on 27 March 2025)	http://ieeexplore.ieee.org/document/10729707/ (accessed on 1 May 2025)	EviPrompt generates training-free evidential prompts for SAM in medical images.
36	SAM-U	SAM-U: Multi-box prompts triggered uncertainty estimation for reliable SAM in medical image	-	https://link.springer.com/chapter/10.1007/978-3-031-47425-5_33 (accessed on 1 May 2025)	Multi-box prompts for an uncertainty-aware SAM in medical images.
37	Ur-sam	Ur-sam: Enhancing the reliability of segment anything model for auto-prompting medical image segmentation with uncertainty rectification	-	https://papers.ssrn.com/sol3/papers.cfm?abstract_id=4878606 (accessed on 1 May 2025)	Uncertainty-rectified SAM for auto-prompting medical segmentation.
38	nnSAM	Plug-and-play Segment Anything Model Improves nnUNet Performance	https://github.com/Kent0n-Li/nnSAM (accessed on 27 March 2025)	https://arxiv.org/pdf/2309.16967 (accessed on 1 May 2025)	nnSAM combines SAM and nnUNet for improved medical image segmentation with limited data.
39	SAMAug	Input Augmentation with SAM: Boosting Medical Image Segmentation with Segmentation Foundation Model	https://github.com/yizhezhang2000/SAMAug (accessed on 27 March 2025)	https://link.springer.com/chapter/10.1007/978-3-031-47401-9_13 (accessed on 1 May 2025)	SAMAug boosts medical image segmentation via input augmentation with the SAM.
40	DB-SAM	DB-SAM: Delving into High Quality Universal Medical Image Segmentation	https://github.com/AlfredQin/DB-SAM (accessed on 27 March 2025)	https://link.springer.com/chapter/10.1007/978-3-031-72390-2_47 (accessed on 1 May 2025)	Dual-branch SAM adapter for universal medical image segmentation.
41	SamDSK	SamDSK: Combining Segment Anything Model with Domain-Specific Knowledge for Semi-Supervised Learning in Medical Image Segmentation	https://github.com/yizhezhang2000/SamDSK (accessed on 27 March 2025)	https://link.springer.com/chapter/10.1007/978-981-97-8496-7_24 (accessed on 1 May 2025)	Combining the SAM with domain knowledge for semi-supervised medical image segmentation.
42	SAM for Semi-Supervised Medical Image Segmentation	Segment anything model for semi-supervised medical image segmentation via selecting reliable pseudo-labels	-	https://link.springer.com/chapter/10.1007/978-981-99-8141-0_11 (accessed on 1 May 2025)	An SAM for selecting reliable pseudo-labels in semi-supervised medical segmentation.
43	SemiSAM	SemiSAM: Enhancing Semi-Supervised Medical Image Segmentation via SAM-Assisted Consistency Regularization	-	https://ieeexplore.ieee.org/abstract/document/10821951 (accessed on 1 May 2025)	SAM-enhanced semi-supervised medical image segmentation.
44	3DSAM-adapter	3DSAM-adapter: Holistic adaptation of SAM from 2D to 3D for promptable tumor segmentation	-	https://www.sciencedirect.com/science/article/pii/S1361841524002494 (accessed on 1 May 2025)	Three-dimensional adapter for the SAM enabling promptable tumor segmentation in volumetric medical images.
45	MA-SAM	MA-SAM: Modality-agnostic SAM Adaptation for 3D Medical Image Segmentation	https://github.com/cchen-cc/MA-SAM (accessed on 27 March 2025)	https://www.sciencedirect.com/science/article/pii/S1361841524002354 (accessed on 1 May 2025)	Modality-agnostic SAM adaptation for 3D medical image segmentation.
46	Promise	Promise: Prompt-driven 3d medical image segmentation using pretrained image foundation models	https://github.com/MedICL-VU/ProMISe (accessed on 27 March 2025)	https://ieeexplore.ieee.org/document/10635207 (accessed on 1 May 2025)	Prompt-driven 3D medical segmentation using a pretrained SAM.
47	Sam3d	Sam3d: Segment anything model in volumetric medical images	https://github.com/UARK-AICV/SAM3D (accessed on 27 March 2025)	https://ieeexplore.ieee.org/document/10635844 (accessed on 1 May 2025)	SAM3D for 3D medical image segmentation.
48	SAM-Med3D	SAM-Med3D: Towards General-purpose Segmentation Models for Volumetric Medical Images	https://github.com/uni-medical/SAM-Med3D (accessed on 27 March 2025)	https://arxiv.org/pdf/2310.15161 (accessed on 1 May 2025)	General-purpose 3D medical image segmentation with promptable SAM-Med3D.
49	Segvol	Segvol: Universal and interactive volumetric medical image segmentation	https://github.com/BAAI-DCAI/SegVol (accessed on 27 March 2025)	https://proceedings.neurips.cc/paper_files/paper/2024/file/c7c7cf10082e454b9662a686ce6f1b6f-Paper-Conference.pdf (accessed on 1 May 2025)	SegVol: Universal 3D medical image segmentation with interactive prompts.
50	SAM+nnUNet	SAM+nnUNet: Deep-learning-based Head and Neck Tumor Segmentation on FDG PET	-	https://ieeexplore.ieee.org/document/10657090 (accessed on 1 May 2025)	SAM+nnUNet improves head and neck tumor segmentation on PET.
51	SAM-UNet	SAM-UNet: Enhancing Zero-Shot Segmentation of SAM for Universal Medical Images	https://github.com/Hhankyangg/sam-unet (accessed on 27 March 2025)	https://arxiv.org/pdf/2408.09886v1 (accessed on 1 May 2025)	SAM-UNet improves medical image segmentation via a U-Net-enhanced SAM.
52	SAM ViT-H D-50 PVTv2 fusion	Improving existing segmentators performance with zero-shot segmentators	https://github.com/LorisNanni/Improving-existing-segmentators-performance-with-zero-shot-segmentators (accessed on 27 March 2025)	https://www.mdpi.com/1099-4300/25/11/1502 (accessed on 1 May 2025)	The SAM enhances existing segmentation models via zero-shot fusion.
54	GazeSAM	GazeSAM: Interactive Image Segmentation with Eye Gaze and Segment Anything Model	https://github.com/ukaukaaaa/GazeSAM (accessed on 27 March 2025)	https://proceedings.mlr.press/v226/wang24a.html (accessed on 1 May 2025)	GazeSAM uses eye-tracking and SAM for automated medical image segmentation.
56	GlanceSeg	GlanceSeg: Real-time microaneurysm lesion segmentation with gaze-map-guided foundation model for early detection of diabetic retinopathy	-	https://ieeexplore.ieee.org/document/10472575/ (accessed on 1 May 2025)	Gaze-guided SAM for real-time diabetic retinopathy lesion segmentation.
57	Uni-COAL	Uni-COAL: A Unified Framework for Cross-Modality Synthesis and Super-Resolution of MR Images	-	https://www.sciencedirect.com/science/article/abs/pii/S0957417424031087 (accessed on 1 May 2025)	Unified framework for MRI cross-modality synthesis and super-resolution.
58	FastSAM-3DSlicer	FastSAM-3DSlicer: A 3D-Slicer Extension for 3D Volumetric Segment Anything Model with Uncertainty Quantification	https://github.com/arcadelab/FastSAM3D_slicer (accessed on 27 March 2025)	https://link.springer.com/chapter/10.1007/978-3-031-73471-7_1 (accessed on 1 May 2025)	3D-Slicer extension for volumetric SAM with uncertainty quantification.
